# Cells of the Maternal–Fetal Interface May Contribute to Epidural-Related Maternal Fever After Administration of Ropivacaine: The Role of Phosphatases DUSP9 and PHLPP1

**DOI:** 10.3390/ijms26125520

**Published:** 2025-06-09

**Authors:** Florian Horn, Verena Tretter, Victoria Kunihs, Peter Wohlrab, Bettina Trimmel, Kevin A. Janes, Tamara Djurkic, Meriem Mekiri, Martin Knöfler, Leila Saleh

**Affiliations:** 1 Clinical Division of General Anesthesia and Intensive Care Medicine, Medical University of Vienna, 1090 Vienna, Austriameriem.mekiri@gesundheitsverbund.at (M.M.); 2 Department of Obstetrics and Gynecology, Reproductive Biology Unit, Placental Development Group, Medical University of Vienna, 1090 Vienna, Austria; 3 Department of Pathology, Medical University of Vienna, 1090 Vienna, Austria; 4 Department of Biomedical Engineering, University of Virginia, Charlottesville, VA 22908, USA

**Keywords:** dual-specificity phosphatase, epidural analgesia, epidural-related maternal fever, DUSP9, PHLPP1, ERMF

## Abstract

Epidural-related maternal fever (ERMF) occurs with significant incidence in women receiving local anesthetics such as ropivacaine via epidural catheter for pain relief during labor. The causal mechanism behind this phenomenon is still not fully resolved, but evidence suggests that these anesthetics cause sterile inflammation. In this observational study, we investigated a possible contributory role of the dual-specificity phosphatase-9 (DUSP9) controlling the activity of mitogen-activated protein kinases (MAPK), and also PH-domain and Leucine-rich repeat phosphatase (PHLPP) regulating AKT kinases. The data show that ropivacaine differentially affects the expression of these phosphatases in distinct cell types of the umbilical cord and placenta. The gene expression of DUSP9 was almost completely switched off in the presence of ropivacaine in HUVECs and extravillous trophoblasts for up to 6 h, while the expression of PHLPP1 was upregulated in HUVECs and syncytiotrophoblasts. Extravillous trophoblasts were identified as a source of pro-inflammatory mediators and regulatory miRNAs in response to ropivacaine. Placentae at term exhibited a distinct DUSP9 expression pattern, whether the patients belonged to the control group or received epidural analgesia with or without elevated body temperature. The observed data imply that ropivacaine induces complex effects on the MAPK and AKT pathways at the feto–maternal interface, which contribute to the ERMF phenomenon.

## 1. Introduction

Epidural analgesia using local anesthetics, with or without the addition of opioids, is primarily applied for pain relief during vaginal delivery. Ropivacaine has several advantages over bupivacaine, such as an improved safety profile and less cardiovascular and nervous system toxicity [1]. The occurrence of intrapartum fever is observed quite frequently and was originally attributed to clinical infections or an extensive inflammatory state at the timepoint of the induction of labor. In 1989, Fusi et al. provided the first evidence of a connection between epidural analgesia and an increase in body temperature [2], a phenomenon that was termed epidural-related maternal fever (ERMF) [3]. The incidence of ERMF varies depending on the type of study and the definition of fever (>37.5 °C, >38 °C, >39 °C) from the single-digit range to almost 50% [3]. As causal explanations were not immediately at hand, ERMF did not receive much further attention until adverse outcomes for mother and child became evident. Several studies have investigated different types of anesthetic delivery, the effect of adjunct opioids, and anesthetic dosing [3]. Meanwhile, it became clear that epidural analgesia is an independent risk factor for intrapartum fever [4]. However, the pathophysiology of this is still insufficiently explored, and several theories have been proposed. As the occurrence of fever is not observed in non-pregnant patients who receive epidural analgesia for surgery, a causal relationship must be linked to the physiology of pregnancy, such as the mechanisms of thermoregulation or an extensive inflammatory state. The latter is a principal feature of labor, but the additional effects of the epidural anesthetic can ultimately lead to a rise in body temperature. Evidence for the latter has been found in several studies showing that ropivacaine induces the release of pyrogenic pro-inflammatory cytokines and prostaglandins from endothelial cells [5] and that bupivacaine reduces leucocyte caspase-1 and plasma levels of the antipyrogenic factor interleukin-1-receptor antagonist (IL-1ra) [6].

In order to further dive into the elucidation of the molecular mechanisms leading to ERMF, we investigated the role of DUSP9 and PHLPP1 in the present study. We followed this path because, in a previous study from our research group [5], we have demonstrated that the mitogen-activated protein kinase (MAPK) signaling and the AKT-kinase pathway were affected by ropivacaine. The DUSP family of phosphatases and PHLPPs, together with other phosphatases, control the activation of these pathways, and DUSP9 is known to have an important functional role in the placenta.

MAPKs are serine/threonine-specific protein kinases that regulate gene expression, proliferation, cell survival/apoptosis, and differentiation, and comprise three families: Extracellular-signal-Regulated Kinase (ERK1/2 and ERK5-BMK pathways), c-Jun N-terminal kinase (JNK), and p38 mitogen-activated protein kinase. These kinases become activated by a cascade of upstream kinase activities, ultimately leading to the phosphorylation of a threonine-x-tyrosine motif. Their activity is swiftly switched off by dephosphorylation of this motif by a number of phosphatases, including serine/threonine, tyrosine, and dual-specificity MAPK phosphatases (MAPK-DUSPs; MKP) [7,8]. The MAPK-DUSP family comprises 10 members, which contain a kinase-interaction motif, in contrast to another group of atypical DUSPs. Class I inducible nuclear MAPK-DUSPs include DUSPs 1, 2, 4, and 5. Class II (DUSPs 6, 7, and 9) are mainly cytoplasmic and have a substrate preference for ERK. Class III (DUSPs 8, 10, and 16) shuttle between the nucleus and the cytoplasm, and dephosphorylate preferably p38 and JNK. Initially, it was believed that members of the MAPK-DUSPs are functionally redundant. However, it became evident that their expression varies in different tissues, that specific subtypes play distinct roles in diseases, and that individual DUSP knock-out mice display characteristic phenotypes. In vitro binding assays show that the preferred MAPK substrate depends on structural details in the binding region [9]. The expression of DUSP genes and the concomitant outcome are not only dependent on extracellular stimuli (ligands) and receptors, but also depend on the cell type.

DUSP9 is expressed from its gene on the X chromosome, and the protein is primarily expressed in the placenta, in addition to the kidney and adipose tissue. DUSP9 seems to be important for placental development and function, as knock-out mice display a phenotype of placental insufficiency [10].

Similar to MAPKs, AKT-kinase—with its three isoforms, AKT1, AKT2, and AKT3—has a central role in cellular metabolism, proliferation, and survival. AKT is sequentially activated by upstream kinases on amino acids Thr-450, Thr-308, and Ser-473 and reciprocally inactivated by PH-domain and Leucine-rich repeat phosphatases (PHLPP1α, PHLPP1/SCOP, and PHLPP2) on Ser-473, protein serine/threonine phosphatase-2A (PP-2A), and others on Thr-308, and by PP-1 on Thr-450 [11]. PHLPP variants play an important role as tumor suppressors in various types of cancers. Both MAPKs and AKT are very important for placental development and function, with significant crosstalk between these signaling pathways [12].

ERMF is a phenomenon only occurring in parturition, which is why we focused on cell types of the placenta, the decidua, and the umbilical cord in this study.

The placenta is a highly complex organ that develops in parallel to the fetus and forms the basis for an intact pregnancy. It represents the connection between mother and child, supplies the fetus with oxygen and nutrients, ensures the removal of metabolic end-products, and guarantees protection of the semi-allogeneic fetus against the maternal immune system [13,14,15]. The epithelial placental cells are called trophoblasts, which can develop differently depending on their localization. The functional units of the placenta are the placental villi, tree-like structures with a villous core consisting of stromal, immune, and endothelial cells surrounded by the bi-layered trophoblast epithelium. Floating placental villi protrude into the intervillous space, where they are in direct contact with the maternal blood. Underlying mononuclear cytotrophoblasts (CTBs) of these villi fuse and form a continuous multinucleated cell layer on the outside, the syncytiotrophoblasts (STs). The STs, bathed in maternal blood, are responsible for molecule transfer from the mother to the child, and vice versa, and the secretion of pregnancy-maintaining hormones, such as human chorionic gonadotrophin [16]. On the other hand, so-called anchoring villi attach to the maternal decidua basalis (DB), the part of the endometrium during pregnancy where implantation takes place. CTBs proliferate here, form cell columns, and gradually detach from the villous tips, migrating into the maternal uterus. This differentiation process gives rise to extravillous trophoblasts (EVTs) and is accompanied by the expression of EVT-specific marker genes, such as HLA-G, and the loss of the proliferative capacity [17,18]. EVTs either invade the decidual stroma as deep as the first third of the myometrium to become multinucleated trophoblast giant cells in that region, or infiltrate and remodel maternal vessels, such as spiral arteries, thereby replacing the endothelial cells to adapt utero–placental blood flow [19,20,21].

In this study, we show that ropivacaine specifically regulates the expression of dual-specificity phosphatase DUSP9 and PHLPP1 in different subtypes of trophoblasts of the human placenta, thereby contributing to an increased inflammatory state, including the release of pyrogens.

## 2. Results

### 2.1. Time-Dependent Regulation of Phosphatase Activities on MAP Kinase ERK and Akt-Kinase by Ropivacaine in HUVECs

**Figure 1:**
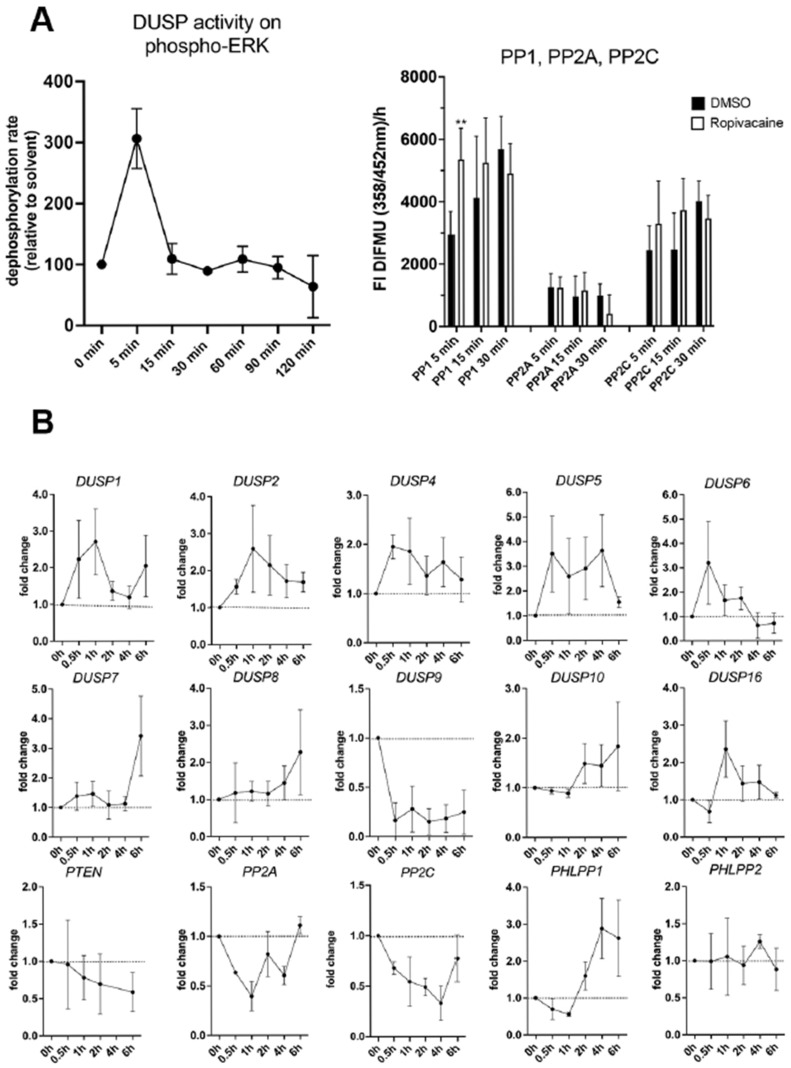
Effects of ropivacaine on different phosphatase activities and expression of MAPK-DUSPs and AKT-regulating phosphatases in HUVECs. (**A**) Phosphatase activity assays of MAPK-DUSPs with ERK as the substrate and protein phosphatases-1, -2A, and -2C in HUVECs upon exposure to 0.01% (100 μg/mL) ropivacaine. (**B**) qPCR analyses of gene expression of MAPK-DUSPs (MKP) and phosphatases regulating the AKT pathway after exposure of HUVECs to 0.01% ropivacaine. The plots show mean values ± S.D. from n = 3 different preparations of HUVECs. 2 Asterisks (**) means *p* smaller than 0.01.

We used human umbilical cord vascular endothelial cells (HUVECs) for our initial experiments, since these cells are of fetal origin and were previously shown to exhibit time-dependent alteration of MAPK and AKT phosphorylation state by ropivacaine [5]. Phosphatase activities were assessed using specific activity assays, as described in the Methods Section (Figure 1A), and mRNA expression was analyzed using qRT-PCR (Figure 1B). Western blot analysis from [5] revealed a transient decrease in ERK phosphorylation to almost undetectable levels and stronger than baseline phosphorylation reoccurring at 120 min of exposure to ropivacaine. AKT was dephosphorylated quickly from 5 min of ropivacaine exposure onwards, with only a little reoccurrence of phosphorylated Ser473 or Thr308, indicating that only a small proportion of AKT protein regained full activation until 240 min. It is likely that these dynamics involve an active role of phosphatases. Therefore, we assessed the activities of different types of phosphatases in the short term and also their mRNA expression in the long term, knowing that their individual effects depend on multiple factors, such as mRNA and protein stability, individual enzymatic activity, and crosstalk between different subtypes. We observed an acute increase in DUSP activity on ERK as the substrate to over 300% of baseline levels and of AKT-regulating phosphatase PP1 to 170% of the baseline levels at 5 min of ropivacaine exposure (Figure 1A). The gene expression of various members of the DUSP family and of phosphatases in the AKT pathway dynamically changed over the full observation period (Figure 1B). All MAPK-DUSPs (MKP) were transcriptionally upregulated until 6 h of ropivacaine exposure, with the exception of DUSP9, which was rapidly downregulated to approximately 10% of the basal expression, remaining low up to 6 h of exposure. The expression of the regulatory phosphatases in the AKT pathway (PTEN, PP2A, and PP2C) was downregulated, with some recovery at 6 h. However, in contrast to its isoform PHLPP2, PHLPP1 became upregulated after 2 h, reaching a maximum of approximately 300% at 4–6 h.

### 2.2. Ropivacaine Differentially Affects the Gene Expression of DUSP1, DUSP9, PHLPP1, and PHLPP2 in Cultured Syncytiotrophoblasts and Extravillous Trophoblasts

**Figure 2:**
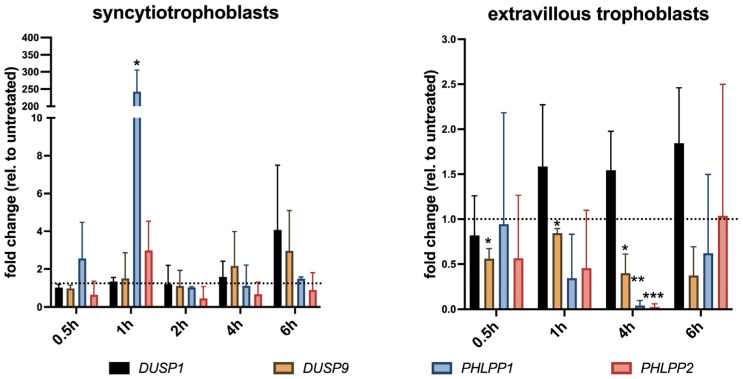
qPCR gene expression analysis for *DUSP1*, *DUSP9*, *PHLPP1*, and *PHLPP2* in cultured syncytiotrophoblasts and extravillous trophoblasts upon exposure to 0.01% ropivacaine. DMSO-stimulated cells served as vehicle controls. Cytotrophoblasts were allowed to fuse for 72 h before stimulation. Non-stimulated controls were arbitrarily set to 1 (= no change; dotted line). Bars indicate mean values ± SD of two isolations (6th to 9th week of pregnancy, n = 4 placentae per isolation), measured in duplicate. * *p* < 0.05; ** *p* < 0.01; *** *p* < 0.001 (Wilcoxon test).

To elucidate the impact of ropivacaine on placental cells, extravillous trophoblasts (EVTs) and cytotrophoblasts (CTBs) were isolated from first-trimester placental tissue. CTBs were allowed to fuse, thereby forming a syncytium-like structure (syncytiotrophoblasts, STs) for 72 h before both EVTs and STs were stimulated with 0.01% ropivacaine for 0.5 h, 1 h, 2 h, 4 h, and 6 h. After the indicated time points, RNA was isolated and qPCR was performed (Figure 2). Despite our primary interest in the DUSP variant DUSP9, we also included DUSP1 as a reference, which is a ubiquitous member of the MAPK-DUSPs. In the STs, *DUSP1* and *DUSP9* mRNA remained unchanged for 2 h, increased after 4 h, and culminated at 6 h, but did not reach statistical significance. In contrast, *PHLPP1* and *PHLPP2* rapidly increased, peaking after one hour, and then returned to basal levels. In EVTs, *DUSP1* mRNA did not show any response to ropivacaine. However, *DUSP9*, *PHLPP1*, and *PHLPP2* continuously regressed in the presence of ropivacaine, while *PHLPP1* and *PHLPP2* levels recovered after 6 h, and *DUSP9* mRNA remained repressed.

### 2.3. Cell-Type-Specific Changes in DUSP9 and PHLPP1 Expression upon Ropivacaine Exposure of First-Trimester Placental Explants

**Figure 3:**
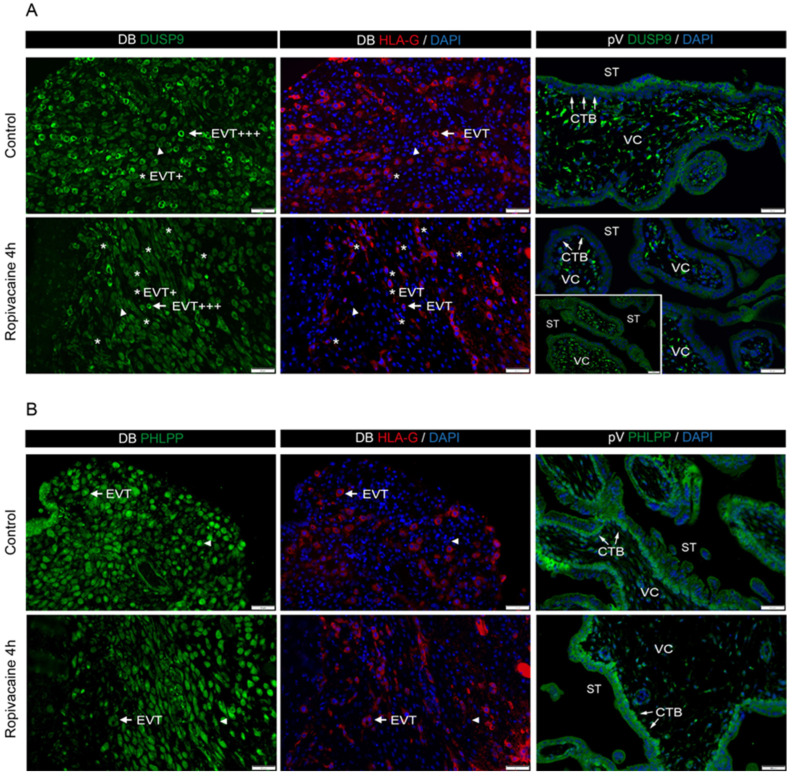
Immunofluorescence analyses showing representative examples of DUSP9 and pan-PHLPP expression in first-trimester decidual and placental explant cultures in the absence (control) or presence of 0.01% ropivacaine. Explants exposed to DMSO served as the vehicle control. (**Left panel**) Representative pictures of DUSP9 (**A**) and pan-PHLPP (**B**) staining (green) of decidua basalis (9th week of pregnancy; n = 2 preparations). Corresponding serial sections stained with HLA-G (red) to visualize EVTs and DAPI (blue; nuclei) are depicted in the **middle panel**. Arrows indicate representative DUSP9- or PHLPP-positive EVTs (HLA-G-positive). Arrowheads mark examples of DUSP9- or PHLPP-positive decidual cells lacking HLA-G expression. Asterisks show representative EVTs with low DUSP9 expression (HLA-G-positive). (**Right panel**) Staining of first-trimester placental villi (9th week of pregnancy; n = 2 preparations) using DUSP9 or PHLPP (**A**,**B**, respectively) antibodies, counterstained with DAPI (blue; nuclei). Arrows indicate examples of cytotrophoblasts. Bars = 50 µm. CTB, cytotrophoblast; DB, decidua basalis; EVTs, extravillous trophoblasts; pV, placental villus; ST, syncytiotrophoblast; VC, villous core.

To get closer to the in vivo situation within a three-dimensional tissue structure containing maternal uterine cells, we investigated ropivacaine stimulation of first-trimester explant cultures of decidua basalis and patient-matched placentae. Immunofluorescence analyses in the control group revealed that most of the HLA-G-positive EVTs were highly positive for DUSP9, and just a few EVTs expressed DUSP9 at a low level (Figure 3A; upper left and middle panel). However, after four hours of stimulation with 0.01% ropivacaine, the number of EVTs highly expressing DUSP9 decreased substantially, while the decidual cells remained unaffected, expressing DUSP9 at a low level (Figure 3A; lower left and middle panel). STs, the cell layer facing the intervillous space, exhibited strong DUSP9 staining in the control group, but lost DUSP9 expression in the presence of ropivacaine (Figure 3A; right panel). However, some villi were detected that still expressed DUSP9 in the STs upon ropivacaine treatment (Figure 3A, lower right panel insert). The villous core (DUSP9-positive) and CTBs (DUSP9-negative), the cell layer underneath the syncytium, did not show a ropivacaine response. EVTs of the decidua basalis were also positive for PHLPP (pan-PHLPP antibody). However, in contrast to DUSP9, this enzyme remained unaffected in EVTs upon ropivacaine treatment. The same was true for decidual cells (Figure 3B; left and middle panel). CTBs of placental villi were PHLPP-positive in the absence and presence of ropivacaine. However, STs, which were PHLPP-negative or displayed weak staining in the controls, became PHLPP-positive after ropivacaine stimulation (Figure 3B; right panel). For the negative controls of IHC staining, the reader is referred to Supplementary Materials S3. A summary of the expression pattern of DUSP9 and PHLPP of the individual cell populations of first-trimester explant cultures is provided in Table 1.

**Figure 4:**
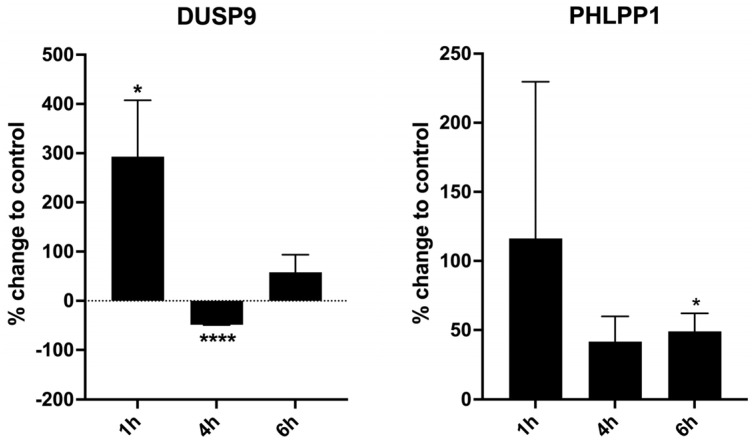
Western blot analysis of first-trimester explant tissue of total decidua basalis exposed to 0.01% ropivacaine for 1 h, 4 h, and 6 h developed with antibodies for DUSP9 and PHLPP1. Tissue samples of similar weight were exposed to 0.01% ropivacaine and DMSO as solvent in parallel. Equal amounts of protein were processed for Western blot analysis. Bars indicate percentage change in ropivacaine-exposed tissue relative to vehicle-treated tissue as mean ± SD. Data are from n = 3 replicates. * *p* < 0.05; **** *p* < 0.0001 (Wilcoxon Test).

Subsequently, native tissue from first-trimester deciduae basalis—exposed to 0.01% ropivacaine for 1 h, 4 h, and 6 h—was processed for Western blot analysis and quantified using a gel imaging system and ImageJ 1.53a software (Figure 4).

In the presence of ropivacaine, decidua basalis tissue, including EVTs, revealed a 3-fold upregulation of DUSP9 at 1 h and an upward trend of PHLPP1. DUSP9 was downregulated by 50% at 4 h of stimulation, while PHLPP1 stayed elevated.

### 2.4. Alterations of miRNA Expression in Cultured Extravillous Trophoblast upon Treatment with Ropivacaine

To obtain insights into the gene regulation of extravillous trophoblasts upon ropivacaine treatment, changes in miRNA expression were analyzed. Cultures of primary extravillous trophoblasts were exposed to 0.01% ropivacaine or solvent (DMSO) for 0.5 h and 4 h, respectively. The quantification of miRNA expression in cell lysates was carried out using small RNA sequencing. miRNAs exhibiting fold changes with *p*-values < 0.05 are shown in Table 2, and miRNAs known to target DUSP9 are shown in Table 3.

**Figure 5:**
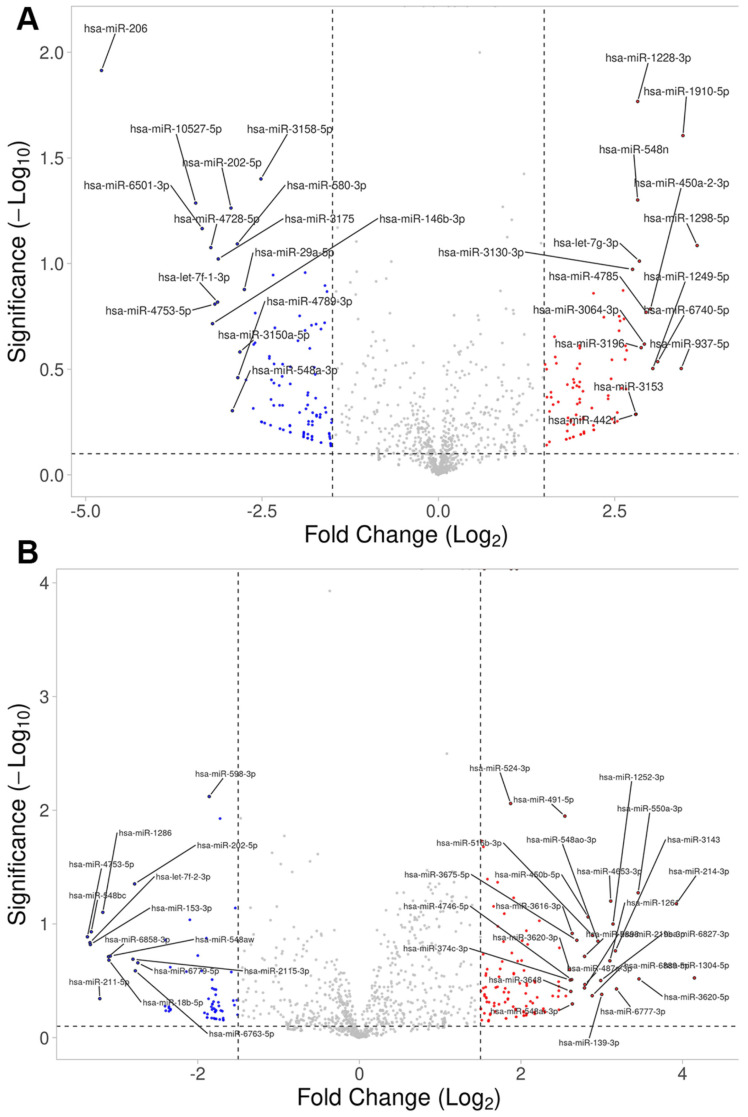
Volcano plots showing altered expressions of miRNAs in extravillous trophoblasts upon ropivacaine exposure. Cells were exposed to 0.01% ropivacaine in the cell culture for (**A**) 0.5 h and (**B**) 4 h. miRNAs were quantified using small RNA sequencing and expressed as log_2_fold change relative to the solvent (DMSO) for the same time span.

Figure 5 shows Volcano plots with upregulated (red) and downregulated (blue) miRNAs after 0.5 h and 4 h of ropivacaine treatment. miRNAs from Table 2 with *p*-values < 0.05 were used for Pathway Enrichment analysis on the platform www.mirnet.com (access of database on 20 February 2025). The enriched terms, according to the Reactome database, are listed in Supplementary Materials S1 and S2.

Enrichment analysis of altered miRNAs after 0.5 h and 4 h of exposure to ropivacaine resulted largely in the identification of overlapping pathways, i.e., (oxidative) stress/heat shock response, apoptosis, alterations in TGFß-SMAD, PI3K-AKT, ERBB4, EGFR, FGFR, NOTCH, Wnt, SCF-KIT, insulin receptor and calcium signaling, the regulation of HIF1α and the hypoxic response, prolonged ERK activation, glucose transport, GLUT4 translocation to the plasma membrane, (NLRP3) inflammasome, interferon signaling, and immune system interaction (for detailed results see Supplementary Materials S1 and S2).

### 2.5. Extravillous Trophoblasts Exhibit an Inflammatory Response upon Exposure to Ropivacaine

**Figure 6:**
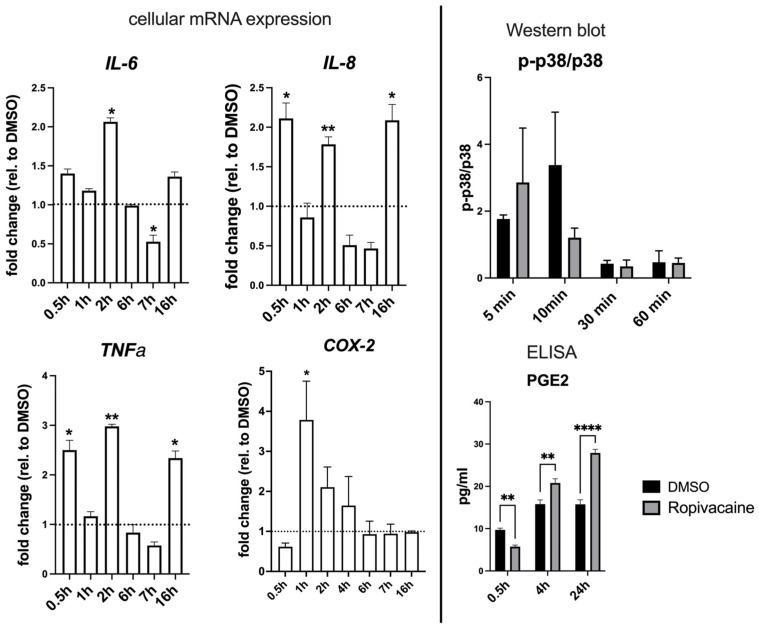
Inflammatory responses in cultured extravillous trophoblasts upon exposure to 0.01% ropivacaine. Due to higher sensitivity, the expression of *TNFα*, *IL-6*, *IL-8*, and *COX-2* was analyzed by qPCR on the mRNA level. The p-p38/p38 ratio was determined from the intensities of the respective bands in Western blots, and PGE2 levels were analyzed in the supernatant by ELISA. Black bars represent treatment with DMSO, grey bars represent treatment with 0.01% ropivacaine in DMSO. All data were determined from n = 3 replicates and are shown as mean ± SD. * *p* < 0.05; ** *p* < 0.01; **** *p* < 0.0001 (two-way ANOVA, multiple comparisons).

In their study, Zhong et al. [22] describe a mechanism in which the downregulation of DUSP9 by miR-132-3p in the amnion ultimately leads to the upregulation of *COX-2* and associated PGE2 release involving p38 signaling. We tested the presence of a similar mechanism in the context of our experimental setup (Figure 6).

As quantitative miRNA and enrichment analyses indicated an inflammatory response of EVTs to ropivacaine treatment, we analyzed the expression of the pro-inflammatory cytokines *IL-6*, *IL-8*, and *TNFα*, as well as the prostaglandin biosynthetic enzyme *COX-2* and PGE2. Increases in these analytes were time-dependent, starting with a significant upregulation of *COX-2* at 1 h and an increase in its product PGE2 after 4 h, until 24 h of ropivacaine exposure. The transcript levels of the cytokines peaked at 0.5 h, 2 h, and 16 h of exposure, while downregulation could be observed at other time points (Figure 6).

### 2.6. Term Placentae from Patients with ERMF Show Distinct Alterations in Cell-Type-Specific DUSP9 Expression

**Figure 7:**
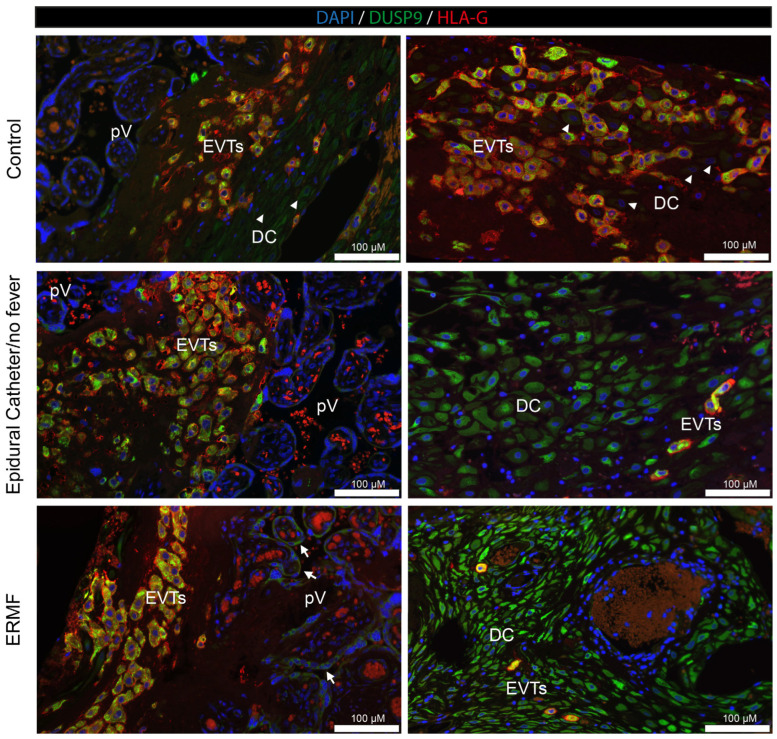
Immunofluorescence analysis of DUSP9 in term placental tissues from women in the absence (control) or presence of an epidural catheter/ropivacaine administration, with or without fever (ERMF and epidural catheter/no fever, respectively). n = 3 per group. Two representative pictures of different parts of the placenta are shown. The **left column** displays decidual tissue in close proximity to the placental villi, and the **right column** shows deeper invasion sites. Sections were stained with DUSP9 (green), HLA-G (red) to visualize EVTs, and DAPI (blue; nuclei). Double-positive EVTs appear orange. Arrows indicated DUSP9-positive syncytiotrophoblasts of placental villi. Arrowheads point towards examples of DUSP9-negative decidual cells. Bars = 100 μm. DC, decidual cells; EVTs, extravillous trophoblasts; pV, placental villi.

Finally, we evaluated term placenta (39th to 40th week of gestation) tissue obtained after parturition from a clinical study, where probands were stratified into a control group (no epidural catheter), epidural catheter/no fever group, and a ERMF group (epidural catheter and elevated body temperature) (n = 3 from each group). Representative immunofluorescence images of serial sections, derived from FFPE term placentae, are shown in Figure 7. We performed DUSP9 immunodetection and counterstained with HLA-G antibodies to discriminate between the decidual cells (HLA-G-negative) and the EVTs (HLA-G-positive). The HLA-G-positive EVTs within the maternal decidua were also positive for DUSP9, resulting in orange staining in all three groups. In contrast, the STs of placental villi were negative for DUSP9 in the controls and patients with an epidural catheter (no fever), but were DUSP9-positive in women suffering from ERMF. The villous core remained DUSP9-negative. The maternal decidual cells did not display DUSP9 staining in the control group. However, a high expression of DUSP9 could be detected in the decidual stromal tissue upon application of ropivacaine, regardless of whether the patients developed a fever or not. Scoring of expression levels of DUSP9 in different cell populations is shown in Table 4.

PHLPP staining was also performed on term placentae, but it did not reveal any differences between the three treatment groups. Each cell population contained cells that were positive and negative for PHLPP1 and PHLPP2.

## 3. Discussion

In this study, we followed up previous investigations with the aim of better understaning the phenomenon of epidural-related maternal fever. The latter represents a challenge in obstetric anesthesiology, since it is suggested to potentially have detrimental consequences for the health of mother and child [23,24].

When epidural analgesia is chosen for pain relief in child birth, the anesthesiologist inserts a catheter into the epidural space for the delivery of local anesthetics such as ropivacaine or bupivacaine, with or without opioids, as needed. In a significant number of patients, the anesthetic induces a rise in body temperature after 4–6 h, which is not caused by the injection technique or some kind of infection, but by effects of the drugs themselves [3]. Interestingly, this observation does not occur in other types of surgeries in non-pregnant patients. In those cases, rather hypothermia frequently develops. Therefore, it is readily apparent that the febrile response in ERMF is most likely due to pregnancy and labor.

Evidence from clinical studies indicates that inflammatory events must be an important part of ERMF, as women with initially higher serum levels of pro-inflammatory cytokine and lower anti-pyrogenic cytokine IL-1ra are more likely to develop ERMF [25]. Also, anti-inflammatory glucocorticoids reduce the incidence of ERMF [26]. Previous in vitro studies have indicated several aspects of sterile inflammation, such as the induction of endogenous pyrogen (IL-6, PGE2) release from vascular cells, the upregulation of cell adhesion molecules for the docking of immune cells, and the release of damage-associated molecular patterns (DAMPs). In addition, oxidative stress, which is at least partly caused by a downregulation of cellular antioxidants, such as superoxide dismutases, thioredoxins, and other ROS-dissipating molecules, could play a role [5]. In a previous study, we also observed that ropivacaine shuts off ERK1/2 and AKT activity for an extended period of time. Both signaling pathways play an important role in the function of the placenta and maintain significant crosstalk [12,27]. The activity of ERK and AKT is determined by their phosphorylation status. These enzymes are activated by upstream kinases and are switched off by different phosphatases. In this study, we focused on the phosphatases DUSPs and PHLPP modulating ERK and AKT activity. The DUSP family is heterogenous, with some substrate preferences, but also has the capability to compensate for other isoforms [28]. PHLPP occurs in three isoforms: PHLPP1α, PHLPP1β/SCOP, and PHLPP2.

We performed initial experiments with HUVECs as a model for vascular endothelial cells of fetal origin. We found that ropivacaine induces a rapid transient increase in DUSP enzymatic activities, as well as PP1 activity, which dephosphorylates AKT in the first place. A specific PHLPP activity assay was not available. Over the next couple of hours, we saw an increase in the mRNA expression of the inducible DUSPs 1, 2, 4, and 5; a later increase in the expression of DUSP6 and 16; and an exceptional decrease to about only 10% of the basal levels in the expression of DUSP9 mRNA from 0.5 h onwards, until at least 6 h. In contrast to PHLPP2, the PHLPP1 isoform was upregulated 3-fold from 2 h onwards.

For several reasons, the drastic downregulation of DUSP9 by ropivacaine caught our specific attention, as DUSP9 has an important role in placental function during gestation, but is dispensable for embryonic development [10]. DUSP9 expression is high in the first-trimester placenta, but it later declines until parturition [29]. DUSP9 levels are significantly lower in the placentae of women with pre-eclampsia [29]. A protective effect against gestational diabetes is also debated, as DUSP9 affects insulin resistance and generally has a complex role in insulin signaling, glucose uptake, and related metabolic processes [30]. DUSP9 is also expressed in several types of blood and immune cells [31]. The immune cell type plasmacytoid dendritic cells express high levels of DUSP9, and its levels seem to correlate with a high production of type I interferon by these cells [32].

In addition to vascular cells, we investigated DUSP9 and PHLPP in placental trophoblasts in the culture and in placental tissue derived from the first trimester of pregnancy. Finally, we analyzed DUSP9 levels in term placentae collected after parturition from women with and without epidural analgesia with ropivacaine. STs and extravillous trophoblasts responded differently to ropivacaine treatment, both in in vitro cultures and in patients suffering from ERMF. Ropivacaine showed a trend to increase DUSP9 mRNA in cultured STs and led to elevated protein levels in the STs of ERMF patients. However, first-trimester placental explant tissues treated with ropivacaine displayed both positive and negative areas of STs, which may be due to additional influences, such as stimuli from neighboring cells.

Similar to HUVECs, primary extravillous trophoblasts responded with a significant downregulation of *DUSP9* mRNA to almost undetectable levels at 4 h of exposure to ropivacaine. This downregulation might be at least partly regulated by miRNAs targeting DUSP9 mRNA, which is a mechanism already found in other contexts. We identified some miRNA candidates that might target DUSP9 in concert; however, due to the small available sample size and the putative heterogeneity of cultures, it did not reach absolute statistical significance. EVTs derive from proliferating cytotrophoblasts, undergo EMT, and start to invade the decidua like tumor cells, where they are either interstitial EVTs or invade the vascular system to remodel spiral arteries and adopt endothelial-like phenotypes. We also observed a decrease in DUSP9 immunostaining in the EVTs from the first-trimester DB explant tissue at 4 h of exposure to ropivacaine. Concordantly, Western blot analyses of the corresponding tissues revealed a downregulation of DUSP9 protein levels in the presence of ropivacaine for 4 h. In term placentae from ERMF patients, the EVTs with strong DUSP9 staining prevailed, and only a few EVTs seemed to be DUSP9-negative. However, it must be mentioned that, in the control group, populations of EVTs with different expression levels of DUSP9 were also identified. Moreover, only the fraction of DB tissue that is attached to the placenta is accessible for investigations. The basal portion of the DB remains in the mother after placental expulsion. Hence, analyses of EVTs at deeper invasion sites are not feasible [33].

*PHLPP1* expression was time-dependently induced by ropivacaine in HUVECs, cultured STs, and on the protein level in the syncytium of the first-trimester placental explants. In cultured EVTs, *PHLPP1* mRNA was transiently reduced, which was not detected on the protein level in the decidua basalis of the first-trimester explants at the investigated timepoints, nor in the Western blots of the corresponding decidual tissues upon ropivacaine treatment.

Cultured EVTs further exhibited a strong upregulation of pro-inflammatory cytokines *IL-6*, *IL-8*, and *TNFα*, as well as *COX-2*, and delayed PGE2 production. Also, in this aspect, these cells respond similarly to HUVECs. The miRNome of the cultured EVTs exposed to ropivacaine displayed a plethora of affected stress-related and inflammatory pathways, as well as signaling pathways typical for EVT signaling.

The upregulation of miRNAs, which repress DUSP9 mRNA, such as miR-1246 and miR-132-3p, has been found previously to be involved in such pro-inflammatory events (see discussion below). DUSP9 expression is regulated on various levels, including transcriptionally, post-transcriptionally, and post-translationally [34,35]. The transcriptional regulation of DUSP9 is known to be mediated by HIF-1α, the EFS family, BMP4 via SMAD1/5, and SMAD4. Post-translational stabilization of DUSP9 has been shown to be mediated by the long non-coding RNA *LincU* [36]. As post-transcriptional regulators, several miRNAs have been described, such as miR-1246, miR-212, miR-133b, miR-4458, and miR-4510 [37].

We analyzed the miRNAome of cultured EVTs, with and without treatment with ropivacaine, for 1 h and 4 h in order to gain some insights into the regulation of DUSP9 and other molecular events occurring in this cell type. The altered miRNAome was analyzed using Pathway Enrichment analysis, which revealed typical pathways known to be affected by ropivacaine treatment (oxidative stress, HIF-1α, AKT signaling, and inflammasome), in the context of DUSP9 regulation (glucose transport, insulin signaling, GLUT4 translocation, interferon signaling, and SMAD transcriptional activity) and EVT signaling (TGFβ-SMAD, NOTCH, Wnt, etc.). In this analysis, we could only include a small sample size, as this material is extremely scarce and ethically restricted. Therefore, we could only work with miRNAs that reached an uncorrected *p*-value of less than 0.05. A closer look at miRNAs with *DUSP9*, as a predicted target gene, revealed a trend of ropivacaine-mediated upregulation for the following miRNAs: miR-1246 (*p* = 0.09), miR-132-3p (*p* = 0.18), miR-3622a-5p (*p* = 0.67), and miR-212-3p (*p* = 0.76)—the last two with already poor statistical quality. DUSP9 regulation by miR-132-3p has recently been described in the context of inflammation. Zhong et al. described that this mechanism putatively mediates the enhancement of inflammation in the amnion involving p38 signaling and increases the expression of COX-2 and PGE2, potentially causing preterm birth [22].

The analysis of term placentae from a clinical study revealed another interesting finding. In this study, probands were stratified into three groups: a control group without an epidural catheter; group 2 with an epidural catheter, but no fever; and group 3, including patients who developed an increased body temperature upon epidural analgesia with ropivacaine. It must be considered that the collection of these placentae occurred many hours after the insertion of the epidural catheter. During this time, the regulation of protein expression can be different compared to that of our observation periods of only up to 6 h. However, the ERMF group showed characteristic details that were different from those of the other groups, since DUSP9 was strongly upregulated in the STs of the placental villi. Moreover, the decidual stroma cells were negative for DUSP9 in the control group but became strongly positive upon the application of ropivacaine via the epidural catheter. The reason for this observation remains unsolved and needs further investigation. Whether these characteristics in the ERMF group are causal to a rise in body temperature or a consequence can currently not be distinguished. Further mechanistic studies of these aspects could include the treatment of naïve term placental tissue with ropivacaine and the consecutive analysis of DUSP9 expression—maybe even on the single cell level. Also, DUSP9 expression could be knocked out or overexpressed in primary tissue to analyze the functional consequences. Animal experiments have already shown that DUSP9 knock-out in the germline is detrimental to labyrinth development and, therefore, placental function [10]. However, the human placenta is anatomically and physiologically different from that of rodents, and conclusions cannot be translated easily.

Apart from its local role in regulating AKT in the placenta, we would like to add another hypothetical aspect of PHLPP1 in this context. PHLPP1β’s alias is SCOP (suprachiasmatic nucleus (SCN) circadian oscillatory protein). The SCN in the ventral hypothalamus is the master regulator of the circadian clock, where PHLPP1β and p-ERK are cyclically upregulated and downregulated, respectively, triggered by light as the stimulus [38]. The circadian clock also regulates the core body temperature. The thermoregulatory center is located in the ventral medial preoptic area of the hypothalamus. Adjacent is found the organum vasculosum of the lamina terminalis (OVLT), which lacks a blood–brain barrier [39]. Therefore, circulatory substances, such as endogenous pyrogens (IL-1, IL-6, TNFα, IFN, and PGE2) and drugs can directly act on the OVLT and raise the body’s core temperature. It is feasible that ropivacaine can reach the OVLT via the blood and could induce changes in PHLPP1 expression in the hypothalamus, thereby resetting the circadian clock, which theoretically could leverage control over the body temperature, resulting in either hyper- or hypothermia. Interestingly, in non-pregnant patients, epidural anesthesia occasionally induces hypothermia. This perspective demands independent investigations, but can serve as a working hypothesis for future studies. The birth process per se goes hand in hand with local inflammatory processes at the feto–maternal interface. Several studies, including our own, have shown that epidural anesthetics additionally enhance the inflammatory state by the release of pro-inflammatory cytokines and prostaglandins (for instance, from the endothelial cells and trophoblasts of the placenta), possibly with additional assistance from the immune system. The already diverse local inflammatory events might sum up with the effects of the anesthetic to pass a threshold in certain individuals, resulting in an elevated body temperature.

## 4. Materials and Methods

### 4.1. Ethics

The utilization of tissues from first trimester legal pregnancy terminations and experimental procedures were approved by the Medical University of Vienna ethics committee (no. 084/2009).

Term placental tissue was obtained from vaginal deliveries from included probands giving birth at the maternity ward of General Hospital Vienna. Written informed consent was obtained from all women donating their placentae for this study. The tissues were processed according to the routine histological examinations by the Department of Pathology, and further use of these data and further analyses were approved by the ethical commission of Medical University of Vienna (no. 2119/2016).

### 4.2. Clinical Study: Patients’ Characteristics

Tissue from term placentae were obtained from the clinical study NCT0405223, registered on clinicaltrials.gov. Details on this study are found in [40]. For immunohistochemistry, we selected paraffine-embedded specimens from 3 probands (patient details inn Table 5) from the control group (no catheter); catheter, no fever group; and ERMF group, respectively, who were representative for their group and displayed no histological abnormalities. The 3 probands of the ERMF group, whose placental tissues were analyzed in this study, developed an increase in body temperature to 37.5 °C, 37.6 °C, and 38 °C, respectively.

### 4.3. Antibodies

All primary and secondary antibodies used in this study for different techniques, like immunohistochemistry and Western blotting are listed in Table 6.

### 4.4. Tissue Collection for Explant Cultures and Immunohistochemistry

First-trimester placental (n = 25) and decidual tissues (n = 3) were obtained from legal pregnancy terminations (sixth to ninth week of gestation). Third-trimester placental tissues (39th to 40th week of gestation, n = 9) were collected upon parturition.

### 4.5. Immunofluorescence of Paraffin-Embedded Tissues

Term placental and first-trimester explant tissues were fixed in 7.5% formaldehyde and embedded in paraffin. Serial sections (3 µm) of paraffin-embedded material were analyzed using immunofluorescence. Sections were deparaffinized in xylol and rehydrated. Antigen retrieval was performed using 1× PT module buffer 1 (ThermoFisher Scientific, Waltham, MA, USA) for 35 min at 93 °C using a KOS microwave Histostation (Milestone). The slides were incubated with primary antibodies (see Table 4) overnight at 4 °C, washed three times with PBS, and subsequently incubated with secondary antibodies (2 µg/mL, Table 4) for one hour. Nuclei were stained with 1 µg/mL DAPI. The tissues were analyzed using fluorescence microscopy (Olympus BX50, Tokyo, Japan) and digitally photographed.

### 4.6. Isolation and Cultivation of Purified Primary Villous Trophoblasts

Primary trophoblasts were isolated by enzymatic digestion of pooled first-trimester placentae (sixth to ninth week of gestation n = 4–7 per isolation; 5 isolations), as recently described, with minor modification [41]. Briefly, placental villi were scraped and digested three times with 0.125% trypsin (Life Technologies, Carlsbad, CA, USA) and 12.5 mg/mL DNase I (Sigma-Aldrich) in Ca^2+^/Mg^2+^-free HBSS (1×HBSS, Sigma-Aldrich) for 15 min at 37 °C. Each digestion step was stopped by adding 10% (vol/vol) FBS (Sigma-Aldrich, St. Louis, MI, USA) and was filtered through a 100 µm cell strainer (ThermoFisher Scientific, Waltham, MA, USA). For the isolation of extravillous trophoblasts (EVTs), the first digestion step was used, while the supernatants of digestions 2 and 3, harboring villous cytotrophoblasts, were pooled. The trophoblasts were enriched with Percoll density gradient centrifugation (Pharmacia, London, UK). Cells between 35% and 50% of the Percoll layers were collected and washed with 1 × HBSS and incubated with erythrocyte lysis buffer (155 mM NH_4_Cl, 10 mM KHCO_3_, 0.1 mM EDTA, pH 7.3) for 5 min at room temperature to remove red blood cells (if present). The EVTs were further enriched by labeling the cells with HLA-G–PE antibody (#1P-292-C100, EXBIO Praha, CZ, final concentration 5 μg/mL) and anti-PE microbeads (Miltenyi Biotec, Bergisch Gladbach, Germany) (#130-048-801, Dilution 1:5), followed by magnetic-activated cell sorting (OctoMACS separator and MS columns, Miltenyi Biotec). Subsequently, the cells were washed with 1 × HBSS, resuspended in culture medium (DMEM/F-12, GlutaMAX, supplemented with 10% FBS Superior, 50 mg/mL gentamicin, and 0.5 mg/mL fungizone), and seeded onto fibronectin-coated (20 µg/mL; MERCK Millipore, Burlington, MA, USA) dishes (2.5 × 10^5^ cells/cm^2^) for 24 h (extravillous trophoblasts) or 72 h (syncytiotrophoblasts, fused cytotrophoblasts). The expression of human chorionic gonadotrophin β (CG-β), a hallmark of the syncytium, and HLA-G, specifying EVTs, was verified using qPCR Thermo Fisher Scientific Predesigned Single Tube TaqMan Gene Expression Assays (CG- β Hs00361224_gH and HLA-G Hs00365950_g1).

### 4.7. Explant Culture

Explants were generated as previously described [42]. Patient-matched placentae and decidua basalis (decB) were cut into small pieces of approximately 1 × 1 cm. The tissues were transferred into 6-well culture dishes and covered with culture medium (DMEM/F-12, GlutaMAX, supplemented with 10% FBS Superior, 50 mg/mL gentamicin, and 0.5 mg/mL fungizone, all ingredients from ThermoFisher Scientific, Waltham, MA, USA), which provides optimal conditions for the viability of the tissue for up to 6 days. Overnight culture was conducted at 37 °C before the tissues were incubated in the absence or presence of 0.01% ropivacaine for different timespans.

For the Western blot experiments, the explants were snap frozen in liquid nitrogen and homogenized in RIPA buffer using Precellys 24 tissue homogenizer (Bertin technologies, Montigny-Le Bretonneux, France).

### 4.8. In Vitro Activity Assays of Dual-Specificity Phosphatases and Serine/Threonine Phosphatases

Plasmids of pGEX-4T-1 containing MEK1 and ERK2, respectively, (GST-3xHA-MEK1-DD and GST-3xFLAG-ERK2) were expressed and purified, essentially as described in [43]. Briefly, the plasmids were transformed into E. coli bl21-DE3 and grown at 37 °C to an optical density of 0.6. IPTG (ß-D-1-thiogalactopyranoside) was used to induce the expression of fusion proteins GST-3xFlag-ERK2 with 2 mM IPTG for 5 h and GST-3xHA-MEK1-DD with 0.4 mM IPTG for 4 h at room temperature. Cells were harvested and lysed in 50 mM Tris pH 7.4, 150 mM NaCl, 1 mM EDTA, 10 μg/mL leupeptin, 10 μg/mL aprotinin, and 1 μg/mL pepstatin in the presence of 0.5 mg/mL lysozyme and 0.5% deoxycholate. Cleared extracts were exposed to glutathione–agarose beads (Sigma, St. Louis, MO, USA), and bound GST-MEK1 was eluted with 10 mM glutathione in 50 mM Tris pH 8.0 and concentrated by ultrafiltration. GST-ERK2 was phosphorylated while being maintained on beads with the concentrated MEK1 in kinase buffer (10 mM Tris pH7.5, 1 mM ATP, 15 mM MgCl_2_, 2.5 mM beta-glycerophosphate, 0.5 mM Na_3_VO_4_, 0.5 mM EGTA, 0.2 mM DTT) for 24 h at 37 °C. Phosphorylated ERK2 was liberated from the beads by digestion with biotinylated thrombin (Thrombin cleavage capture kit; Novagen-MERCK, Birmingham, UK) for 2 h at room temperature. For this purpose, 50 mL beads (50% slurry) with bound fusion protein were incubated with 10 mL 10× thrombin cleavage buffer, 2 mL enzyme (stock diluted 1:25), and 38 mL H_2_O. After digestion, the enzyme was removed with 20 mL streptavidin–agarose. For quality control, fusion protein expression and purification were surveilled via SDS-gel electrophoresis and Western blot analysis.

For the in vitro phosphatase assay, as described in [43], we used high-protein-binding polystyrene microtiter plates (Greiner bio-one, Kremsmünster, Austria) that were coated with recombinant 100 μL phosphorylated ERK2 overnight at 4 °C. The optimal concentration of phosphorylated fusion protein for coating was determined by ELISA as a protein amount resulting in 80% maximum signal saturation (=16 ng/well phosphorylated ERK2). After overnight incubation, the wells were washed three times with 300 μL PBS, 0.1% Tween-20. In order to determine DUSP activity in cell lysates, we added lysate, prepared as in [43], from an equivalent of approximately 25,000 cells per well and incubated it at 30 °C for 30 min. The reaction was terminated with 90 μL phosphatase inhibitor buffer (20 mM Na_4_P_2_O_7_, 60 mM NaF, 400 mM Na_3_VO_4_). The plates were washed and blocked for 1 h with 5% BSA in PBS, 0.1% Tween-20 at room temperature. The detection of the remaining phosphorylated epitopes was performed with anti-phospho-p44/p42 (Cell Signaling Technologies, Danvers, MA, USA; 1:1000). After a further washing step, incubation with secondary biotinylated goat anti-rabbit antibody (Jackson Immunoresearch, West Grove, PA, USA; 1:10.000) was performed for 1 h at room temperature. The plates were washed and incubated with streptavidin-HRP (R&D Systems, Minneapolis, MN, USA; 1:200) for 30 min at room temperature. After a further wash, color development was obtained with the HRP substrate TMB (3,3′,5,5′-tetramethylbenzidine) by incubation for 30 min, after which the reaction was stopped with 50 μL 2N H_2_SO_4_. The optical density was read on a Victor 3 spectrophotometer at 450 nm, with background correction at 540 nm.

Activities of serine/threonine phosphatases PP1, PP2A, and PP2C were assessed using the RediPlate 96 EnzCheck Serine/Threonine Phosphatase Assay from Invitrogen (Waltham, MA, USA), according to the manufacturer’s instructions.

### 4.9. Western Blot Analysis

After protein concentrations were assessed using a BCA protein assay (PIERCE, Waltham, MA, USA), proteins from cell or tissue lysates were precipitated, according to the method of Wessel and Fluegge [44]. Equal amounts of proteins were dissolved in SDS sample buffer and run on SDS-polyacrylamide gels, according to the procedure described by Neville and Glossmann [45]. Protein bands were semi-dry blotted onto nitrocellulose membranes (Millipore, Burlington, MA, USA) and developed using primary and secondary antibodies, as listed in Table 1, followed by chemiluminescence detection using the WesternBright Sirius chemiluminescence substrate (Biozym, Hessisch Oldendorf, Germany) and visualization on a VilberLourmat gel imaging system.

### 4.10. Quantitative Real-Time PCR

mRNA was isolated from cell lysates using the RNEasy mini Plus Kit (Qiagen, Hilden, Germany), and 500 ng was reverse transcribed using the cDNA superscript Kit (Quanta Biosciences, Beverly, MA, USA). Amplification was performed using perfeCTa SYBR Green Fast Mix (Quanta Biosciences, Beverly, MA, USA) on a Rotor Gene Q (Qiagen, Hilden, Germany) using the following temperature program: 3 min 95 °C; and 45 cycles, 30 s 95 °C, 15 s 56 °C, 10 s 72 °C. Primer sequences are listed in Table 7.

### 4.11. Small RNA Sequencing

Sequencing libraries were prepared at the Core Facility Genomics, Medical University of Vienna (a member of VLSI) using the QIAgen smallRNA Library Prep Kit with Unique Molecular Identifiers (UMIs) and RNA Unique Dual Indices (RUDIs), according to the manufacturer’s protocols. The libraries were QC-checked on a Bioanalyzer 2100 (Agilent, Santa Clara, CA, USA) using a High-Sensitivity DNA Kit for the correct insert size and quantified using Qubit dsDNA HS Assay (Invitrogen). Pooled libraries were sequenced on a NextSeq2000 instrument (Illumina) in 1 x 72 bp single-end sequencing mode. Sequencing data were demultiplexed without adapter trimming or short-read masking. On average, 9.6 million reads were generated per sample. Reads in the fastq format were aligned to miRBase_v22 and Homo sapiens (GRCh38.103) and quantified considering the UMIs using the QIAgen GeneGlobe RNA-seq Analysis Portal 4.0 (link accessed 15 June 2023), https://geneglobe.qiagen.com/at/analyze/. Read counts per miRNA were downloaded from GeneGlobe, and differential miRNA expression was calculated using DESeq2 [46] version 1.22.2.

### 4.12. Statistical Methods

Statistical calculations were performed with GraphPad Prism Software version 10.4.1. The Student’s *t*-test was used to compare two groups of normal distributed data. The Wilcoxon test was used as a non-parametric test to test statistical differences between the median of the data and a reference value. To compare the means of two or more groups with two independent factors, a two-way ANOVA was used. The statistical tests used in the individual experiments are indicated in the figure legends.

## 5. Conclusions

In this study, we used a combination of in vitro cell culture and tissue culture experiments, as well as specimens from a clinical study, to investigate the roles of DUSP9 and PHLPP in the maternal–fetal interface that might contribute to the clinical phenomenon of epidural-related maternal fever. These phosphatases respond strongly, and in a cell-type-specific way, to ropivacaine, thereby putatively disturbing the balanced regulation of the MAPK and AKT pathways, which both play an important role in ininflammation. Our results have revealed extravillous trophoblasts as an additional source of pro-inflammatory mediators that might exaggerate the already prevalent inflammatory state at the end of gestation. Additional independent contributing factors might lead to a rise in body temperature as the final outcome.

## Figures and Tables

**Table 1:** Evaluation of immunofluorescence analyses of first-trimester decidual and placental explants. Scoring was performed by two different investigators. +++ very high, ++ high, + low, − negative.

Protein	Cell Population	Control	Ropivacaine 4 h
DUSP9	EVT	+++/+	Decreased number of +++
	Villous core	+++	No change
	Decidual cells	+	No change
	Syncytiotrophoblast	++	++/−
	Cytotrophoblast	−	No change
PHLPP	EVT	+++	No change
	Villous core	++	No change
	Decidual cells	+++	No change
	Syncytiotrophoblast	+/−	+++
	Cytotrophoblast	+++	No change

**Table 2:** Changes in miRNA gene expression in response to the exposure of extravillous trophoblasts with 0.01% ropivacaine for 0.5 h and 4 h, respectively. miRNAs with uncorrected *p*-values < 0.05 are listed. Fold changes were calculated relative to the solvent (DMSO) controls, which were performed in parallel to ropivacaine exposure for 0.5 h and 4 h, respectively, with cells from the same batch. Data are from n = 3 biological replicates.

	miRNA	Log_2_Fold Change (lfcSE)	*p*-Value
0.5 h	hsa-miR-378-5p	0.586 (0.227)	0.010
	hsa-miR-206	−4.776 (1.905)	0.012
	hsa-miR-1228-3p	2.825 (1.184)	0.017
	hsa-miR-910-5p	3.467 (1.544)	0.024
	hsa-miR-365b-5p	1.213 (0.583)	0.037
	hsa-miR-3158-5p	−2.515 (1.223)	0.039
	hsa-miR-548n	2.825 (1.441)	0.050
4 h	hsa-miR-4455	−0.366 (0.095)	0.0001
	hsa-miR-652-3p	1.083 (0.367)	0.003
	hsa-miR-598-3p	−1.858 (0.695)	0.007
	hsa-miR-524-3p	1.872 (0.713)	0.008
	hsa-miR-491-5p	2.543 (1.003)	0.011
	hsa-miR-6515-5p	−1.469 (0.583)	0.011
	hsa-miR-653-3p	−1.724 (0.685)	0.011
	hsa-miR-590-5p	−0.928 (0.388)	0.016
	hsa-miR-512-3p	1.511 (0.642)	0.018
	hsa-miR-106b-3p	1.465 (0.624)	0.018
	hsa-miR-525-3p	1.535 (0.665)	0.021
	hsa-miR-579-3p	−1.079 (0.477)	0.023
	hsa-miR-378a-5p	−0.514 (0.228)	0.024
	hsa-miR-19a-3p	−0.609 (0.277)	0.028
	hsa-miR-514a-5p	−0.972 (0.447)	0.029
	hsa-miR-365a-5p	0.838 (0.394)	0.033
	hsa-miR-4485-3p	1.152 (0.545)	0.034
	hsa-miR-372-5p	−0.831 (0.394)	0.034
	hsa-miR-20a-5p	1.077 (0.520)	0.038
	hsa-miR-135b-5p	1.585 (0.773)	0.040
	hsa-miR-519c-3p	1.171 (0.579)	0.042
	hsa-let-7e-3p	1.710 (0.845)	0.043
	hsa-miR-28-5p	0.873 (0.433)	0.044
	hsa-miR-520c-3p	0.986 (0.490)	0.044
	hsa-miR-202-5p	−2.781 (1.384)	0.044
	hsa-miR-320b	0.943 (0.475)	0.047
	hsa-miR-31-5p	0.853 (0.430)	0.047

**Table 3:** Changes in miRNA abundance targeting *DUSP9* in response to the exposure of extravillous trophoblasts with 0.01% ropivacaine for 4 h. Listed are miRNAs with uncorrected *p*-values < 0.05.

miRNA	Fold Change	*p*-Value
hsa-miR-3622a-5p	3.51	0.67
hsa-miR-212-3p	1.83	0.76
hsa-miR-132-3p	1.98	0.18
hsa-miR-1246	1.54	0.09
hsa-miR-4667-5p	0.23	0.26

**Table 4:** Evaluation of DUSP9 immunofluorescence of term placenta and decidua. Scoring was performed by two different investigators. +++ very high, ++ high, − negative.

Protein	Cell Population	Control	Epidural Catheter/ No Fever	ERMF
DUSP9	EVT	+++	+++	+++
	Villous core	−	−	−
	Decidual cells	−	++	+++
	Syncytiotrophoblasts	−	−	++

**Table 5:** Patient data associated with term placentae investigated in this study.

Patient/Group	Age (Years)	Height (cm)	Weight (kg)
Control	22	165	75
	26	170	100
	21	175	80
Epidural catheter, no fever	41	167	91
	34	175	65
	28	160	92
ERMF	26	158	68
	25	172	83
	31	160	57

**Table 6:** Antibodies used for immunohistochemistry and Western blot analysis.

Antibody	Company/cat.nr.		Species	Dilution IHC	Dilution Western Blot
HLA-G	NOVUS Biologicals ^1^	NB500-302	Mouse	1:200	
DUSP9	Invitrogen ^2^	AB_2854196	Rabbit	1:400	1:400
PHLPP (pan)	Proteintech ^3^	22789-1-AP	Rabbit	1:400	
PHLPP1	Gene Tex ^4^	GTX64568	Rabbit		1:500
PHLPP2	Gene Tex ^4^	GTX31862	Rabbit		1 μg/mL
Anti-rabbit 488	Invitrogen ^2^	A11011	Goat	1:1000	
Anti-mouse 568	Invitrogen ^2^	A11019	Goat	1:1000	
Anti-rabbit IgG(H&L)-HRP	Rockland ^5^	A11017	Goat		1:2000
Anti-mouseIgG(H&L)-HRP	Rockland ^5^	A21069	Goat		1:1000
Mouse IgG1 isotype control	Exbio ^7^	11-457-C100	Mouse	1:200	1:5000
mAb IgG XP R isotype control	Cell Signaling Technologies ^6^	3900	Rabbit	1:400	1:5000

^1^ Novus Biologicals, Centennial, CO, USA; ^2^ Invitrogen, Waltham, MA, USA. ^3^ Proteintech, Rosemont, IL, USA. ^4^ Gene Tex, Irvine, CA, USA. ^6^ Cell Signaling Technologies, Danvers, MA, USA. ^5^ Rockland, Philadelphia, PA, USA. ^7^ Exbio, Prague, Czech Republic.

**Table 7:** Primers used for qRT-PCR.

Gene	Forward Primer	Reverse Primer	Tm
*Dusp1*	CTGCCTTGATCAACGTCTCA	CTGTGCCTTGTGGTTGTCCT	57.3 °C/59.4 °C
*Dusp2*	TGCCCCAACCACTTTGAGG	AGTCAATGAAGCCTATGGCCT	58.8 °C/57.9 °C
*Dusp4*	GGCGGCTATGAGAGGTTTTCC	TGGTCGTGTAGTGGGGTCC	61.8 °C/61.0 °C
*Dusp5*	GCCAGCTTATGACCAGGGTG	GTCCGTCGGGAGACATTCAG	61.4 °C/61.4 °C
*Dusp6*	GAAATGGCGATCAGCAAGACG	CGACGACTCGTATAGCTCCTG	59.8 °C/61.8 °C
*Dusp7*	GACGTGCTCGGCAAGTATG	GGATCTGCTTGTAGGTGAACTC	58.8 °C/60.3 °C
*Dusp8*	TCAGCTCCGTCAACATCTGC	CGCGTGCTCTGGTCATAGA	59.4 °C/58.8 °C
*Dusp9*	CAGCCGTTCTGTCACCGTC	CAAGCTGCGCTCAAAGTCC	61.0 °C/58.8 °C
*Dusp10*	ATCGGCTACGTCATCAACGTC	TCATCCGAGTGTGCTTCATCA	59.8 °C/57.9 °C
*Dusp16*	GCCCATGAGATGATTGGAACTC	CGGCTATCAATTAGCAGCACTTT	60.3 °C/58.9 °C
*Phlpp1*	GTTCTGCCACTAATTGGTGGA	GCTGGGATGCAACCTTGGA	57.9 °C/58.8 °C
*Phlpp2*	TGGAACCTACTGAACGACCTC	ATCCAAACGATCCATGTGGCA	59.8 °C/57.9 °C
*Pten*	TGGATTCGACTTAGACTTGACCT	GGTGGGTTATGGTCTTCAAAAGG	58.9 °C/60.6 °C
*PP2A*	TCTCAGGCATACGCTGACTAC	GGAGACTCTGTACTCGAAGGT	59.8 °C/59.8 °C
*Actb*	CATGTACGTTGCTATCCAGGC	CTCCTTAATGTCACGCACGAT	59.8 °C/57.9 °C

## Data Availability

Original raw data can be obtained from the authors upon request.

## Supplementary Materials

The following supporting information can be downloaded at: https://www.mdpi.com/article/10.3390/ijms26125520/s1.

## Abbreviations

The following abbreviations are used in this manuscript:

AKT	Protein kinase B
CTB	Cytotrophoblast
DB	Decidua basalis
DUSP	Dual-specificity phosphatase
EGFR	Epidermal growth factor receptor
ERBB	Epidermal growth factor
ERMF	Epidural-related maternal fever
EVT	Extravillous trophoblast
FGFR	Fibroblast growth factor receptor
GST	Glutathione S-transferase
HLA-G	Human leucocyte antigen-G
HUVECs	Human umbilical vein endothelial cells
IFN	Interferon
IHC	Immunohistochemistry
IL	Interleukin
qRT-PCR	Quantitative real-time polymerase chain reaction
MAPK	Mitogen-activated protein kinase
MKP	MAPK-phosphatase
NOTCH	Neurogenic locus notch homolog protein
PHLPP	PH-domain and Leucine-rich repeat phosphatase
PI3K	Phosphoinositid-3-kinase
PP1, 2A, 2C	Protein phosphatase 1, 2A, 2C
pV	Placental Villus
SCF	Stem Cell Factor
SMAD	Suppressor of Mothers against Decapentaplegic
ST	Syncytiotrophoblast
TGF	Transforming growth factor
VC	Villous Core
Wnt	Wingless/Integrated
