# Supplementary material for: Cells of the Maternal–Fetal Interface May Contribute to Epidural-Related Maternal Fever After Administration of Ropivacaine: The Role of Phosphatases DUSP9 and PHLPP1
Source: Int J Mol Sci. 2025 Jun 9;26(12):5520. doi: 10.3390/ijms26125520 (PMC12193418; doi:10.3390/ijms26125520)
Supplement: Supplementary file 1 [file ijms-26-05520-s001.zip › ijms-3620049-supplementary/Supplementary Material/Supplementary Material S1 (0.5h Ropivacaine).pdf]

mirnet_enrichment-2

Pathway	Total	Expected	Hits	Pval	FDR
Post-transcriptional silencing by small RNAs	7	154	3	0.000339	0.0153
Oncogene Induced Senescence	30	658	5	0.000434	0.0153
Cellular responses to stress	256	5.61	15	0.000458	0.0153
Ca2+ activated K+ channels	9	197	3	0.000788	0.0197
Regulation of HSF1-mediated heat shock response	54	1.18	6	0.00109	0.0218
Regulatory RNA pathways	100	2.19	8	0.0015	25
Viral Messenger RNA Synthesis	27	592	4	0.00263	0.0295
Gene Expression	851	18.7	31	0.00269	0.0295
Influenza Viral RNA Transcription and Replication	113	2.48	8	0.00325	0.0295
Regulation of Glucokinase by Glucokinase Regulatory Protein	29	636	4	0.00344	0.0295
Influenza Life Cycle	117	2.57	8	0.00402	0.0295
Rev-mediated nuclear export of HIV RNA	31	0.68	4	0.00441	0.0295
Nuclear import of Rev protein	31	0.68	4	0.00441	0.0295
Vpr-mediated nuclear import of PICs	31	0.68	4	0.00441	0.0295
Cellular response to heat stress	72	1.58	6	0.00478	0.0295
Nuclear Pore Complex (NPC) Disassembly	32	702	4	0.00495	0.0295
Influenza Infection	122	2.68	8	0.00518	0.0295
Interactions of Rev with host cellular proteins	33	724	4	0.00554	0.0295
Synthesis of glycosylphosphatidylinositol (GPI)	17	373	3	0.00561	0.0295
Interactions of Vpr with host cellular proteins	34	746	4	0.00618	0.0309
Mitotic Prophase	77	1.69	6	0.00663	0.0316
Intrinsic Pathway for Apoptosis	36	789	4	0.00758	0.0337
Pre-NOTCH Transcription and Translation	19	417	3	0.00774	0.0337
Apoptotic factor-mediated response	7	154	2	0.00933	0.0389
Transcriptional activation of mitochondrial biogenesis	21	461	3	0.0103	0.0397
Ca2+ pathway	40	877	4	11	0.0397
Glucose transport	40	877	4	11	0.0397
beta-catenin independent WNT signaling	113	2.48	7	0.0118	0.0397
Regulation of mRNA stability by proteins that bind AU-rich elements	87	1.91	6	0.0118	0.0397
Hexose transport	42	921	4	13	0.0397
Nuclear Envelope Breakdown	42	921	4	13	0.0397
Cellular Senescence	143	3.14	8	13	0.0397
Eukaryotic Translation Initiation	116	2.54	7	0.0135	0.0397
Cap-dependent Translation Initiation	116	2.54	7	0.0135	0.0397
ISG15 antiviral mechanism	66	1.45	5	0.0145	0.0403
Antiviral mechanism by IFN-stimulated genes	66	1.45	5	0.0145	0.0403
Pre-NOTCH Expression and Processing	24	526	3	0.0149	0.0403
Post-translational modification: synthesis of GPI-anchored proteins	26	0.57	3	0.0186	48
Regulation of signaling by NODAL	10	219	2	0.0192	48
Regulation of the Fanconi anemia pathway	10	219	2	0.0192	48

GAB1 signalosome	98	2.15	6	0.0203	0.0495
Transcriptional regulation by small RNAs	73	1.6	5	0.0216	0.0513
MyD88 deficiency (TLR2/4)	11	241	2	0.0231	0.0513
IRAK4 deficiency (TLR2/4)	11	241	2	0.0231	0.0513
Purine ribonucleoside monophosphate biosynthesis	11	241	2	0.0231	0.0513
Metabolism of proteins	645	14.1	22	0.0237	0.0515
Mitochondrial biogenesis	30	658	3	0.0272	0.0579
L13a-mediated translational silencing of Ceruloplasmin expression	108	2.37	6	0.0309	0.0617
3' -UTR-mediated translational regulation	108	2.37	6	0.0309	0.0617
Transcriptional Regulation by TP53	55	1.21	4	0.0319	0.0617
TP53 Regulates Metabolic Genes	55	1.21	4	0.0319	0.0617
GTP hydrolysis and joining of the 60S ribosomal subunit	109	2.39	6	0.0321	0.0617
Post-translational protein modification	309	6.78	12	0.0383	0.0679
Mitochondrial protein import	35	768	3	0.0406	0.0679
Translation	146	3.2	7	41	0.0679
Signalling to p38 via RIT and RIN	15	329	2	0.0416	0.0679
Signaling by Wnt	245	5.37	10	0.0424	0.0679
Oxidative Stress Induced Senescence	88	1.93	5	0.0435	0.0679
Metabolism of carbohydrates	247	5.42	10	0.0444	0.0679
Cell Cycle	498	10.9	17	0.0445	0.0679
ARMS-mediated activation	16	351	2	0.0469	0.0679
G2/M DNA damage checkpoint	16	351	2	0.0469	0.0679
Homologous Recombination Repair	16	351	2	0.0469	0.0679
Homologous recombination repair of replication-independent double-stra	16	351	2	0.0469	0.0679
Signaling by FGFR	151	3.31	7	0.0477	0.0679
Signaling by FGFR1	151	3.31	7	0.0477	0.0679
Signaling by FGFR2	151	3.31	7	0.0477	0.0679
Signaling by FGFR3	151	3.31	7	0.0477	0.0679
Signaling by FGFR4	151	3.31	7	0.0477	0.0679
Signaling by ERBB2	152	3.33	7	0.0492	0.0679
Butyrate Response Factor 1 (BRF1) destabilizes mRNA	17	373	2	0.0524	0.0679
Tristetraprolin (TTP) destabilizes mRNA	17	373	2	0.0524	0.0679
Branched-chain amino acid catabolism	17	373	2	0.0524	0.0679
Immune System	942	20.7	28	0.0546	0.0679
PI3K events in ERBB4 signaling	94	2.06	5	55	0.0679
PIP3 activates AKT signaling	94	2.06	5	55	0.0679
PI3K events in ERBB2 signaling	94	2.06	5	55	0.0679
PI-3K cascade:FGFR1	94	2.06	5	55	0.0679
PI-3K cascade:FGFR2	94	2.06	5	55	0.0679
PI-3K cascade:FGFR3	94	2.06	5	55	0.0679
PI-3K cascade:FGFR4	94	2.06	5	55	0.0679
Host Interactions of HIV factors	126	2.76	6	0.0581	0.07

Frs2-mediated activation	18	395	2	0.0581	0.07
PI3K/AKT activation	97	2.13	5	0.0614	0.0727
Formation of a pool of free 40S subunits	98	2.15	5	0.0636	0.0727
Signaling by NODAL	19	417	2	64	0.0727
RNA Polymerase III Chain Elongation	19	417	2	64	0.0727
RNA Polymerase III Transcription Termination	19	417	2	64	0.0727
Downstream signaling events of B Cell Receptor (BCR)	164	3.6	7	0.0684	0.0769
Prolonged ERK activation events	20	439	2	0.0701	0.0779
Signaling by SCF-KIT	133	2.92	6	0.0716	0.0787
M Phase	235	5.15	9	0.0727	79
Role of LAT2/NTAL/LAB on calcium mobilization	103	2.26	5	0.0753	0.0804
Signaling by EGFR	168	3.68	7	0.0756	0.0804
Double-Strand Break Repair	21	461	2	0.0765	0.0805
SUMOylation of DNA damage response and repair proteins	74	1.62	4	0.0787	0.0811
SUMO E3 ligases SUMOylate target proteins	74	1.62	4	0.0787	0.0811
Downstream signaling of activated FGFR1	139	3.05	6	0.0845	0.0845
Downstream signaling of activated FGFR2	139	3.05	6	0.0845	0.0845
Downstream signaling of activated FGFR3	139	3.05	6	0.0845	0.0845
