# Supplementary material for: Cells of the Maternal–Fetal Interface May Contribute to Epidural-Related Maternal Fever After Administration of Ropivacaine: The Role of Phosphatases DUSP9 and PHLPP1
Source: Int J Mol Sci. 2025 Jun 9;26(12):5520. doi: 10.3390/ijms26125520 (PMC12193418; doi:10.3390/ijms26125520)
Supplement: Supplementary file 1 [file ijms-26-05520-s001.zip › ijms-3620049-supplementary/Supplementary Material/Supplementary Material S2 (4h ropivacaine).pdf]

mirnet_enrichment

Pathway	Total	Expected	Hits	Pval	FDR
Gene Expression	851	61.7	104	1.68e-08	1.68e-06
Cellular responses to stress	256	18.5	41	8.88e-07	4.44e-05
Downregulation of SMAD2/3:SMAD4 transcriptional activity	22	1.59	9	1.08e-05	0.00036
Signaling by ERBB4	143	10.4	25	2.85e-05	0.000538
Oncogene Induced Senescence	30	2.17	10	2.91e-05	0.000538
Generic Transcription Pathway	189	13.7	30	3.23e-05	0.000538
Signaling by TGF-beta Receptor Complex	72	5.22	16	4.16e-05	0.000594
GAB1 signalosome	98	7.1	19	6.14e-05	0.000683
PI3K events in ERBB4 signaling	94	6.81	18	0.000113	0.000683
PIP3 activates AKT signaling	94	6.81	18	0.000113	0.000683
PI3K events in ERBB2 signaling	94	6.81	18	0.000113	0.000683
PI-3K cascade:FGFR1	94	6.81	18	0.000113	0.000683
PI-3K cascade:FGFR2	94	6.81	18	0.000113	0.000683
PI-3K cascade:FGFR3	94	6.81	18	0.000113	0.000683
PI-3K cascade:FGFR4	94	6.81	18	0.000113	0.000683
Immune System	942	68.3	97	0.000122	0.000683
Oxidative Stress Induced Senescence	88	6.38	17	0.000156	0.000683
Oxygen-dependent proline hydroxylation of Hypoxia-inducible Factor Alpha	18	1.3	7	0.000158	0.000683
Signaling by EGFR	168	12.2	26	0.000166	0.000683
PI3K/AKT activation	97	7.03	18	0.000172	0.000683
Transcriptional activity of SMAD2/SMAD3:SMAD4 heterotrimer	43	3.12	11	0.000176	0.000683
Fc epsilon receptor (FCERI) signaling	169	12.2	26	0.000183	0.000683
Fanconi Anemia pathway	24	1.74	8	0.000187	0.000683
Signaling by FGFR	151	10.9	24	0.000194	0.000683
Signaling by FGFR1	151	10.9	24	0.000194	0.000683
Signaling by FGFR2	151	10.9	24	0.000194	0.000683
Signaling by FGFR3	151	10.9	24	0.000194	0.000683
Signaling by FGFR4	151	10.9	24	0.000194	0.000683
Signaling by SCF-KIT	133	9.64	22	0.000198	0.000683
Signaling by ERBB2	152	11	24	0.000215	0.000717
Regulation of Hypoxia-inducible Factor (HIF) by oxygen	25	1.81	8	0.000257	0.000803
Cellular response to hypoxia	25	1.81	8	0.000257	0.000803
TGF-beta receptor signaling activates SMADs	32	2.32	9	0.000317	0.000961
Downregulation of TGF-beta receptor signaling	26	1.88	8	0.000349	0.00103
Role of LAT2/NTAL/LAB on calcium mobilization	103	7.46	18	0.000374	0.00105
Regulation of HSF1-mediated heat shock response	54	3.91	12	0.000378	0.00105
Downstream signal transduction	151	10.9	23	0.000493	0.00133
Cellular Senescence	143	10.4	22	0.000565	0.00149
DAP12 signaling	154	11.2	23	0.000655	0.00168
Downstream signaling events of B Cell Receptor (BCR)	164	11.9	24	0.00068	0.0017
ISG15 antiviral mechanism	66	4.78	13	0.000752	0.00174
Antiviral mechanism by IFN-stimulated genes	66	4.78	13	0.000752	0.00174
RNA Polymerase I, RNA Polymerase III, and Mitochondrial Transcription	66	4.78	13	0.000752	0.00174

Loss of Function of SMAD2/3 in Cancer	7	507	4	0.000799	0.00174
SMAD2/3 MH2 Domain Mutants in Cancer	7	507	4	0.000799	0.00174
Post-transcriptional silencing by small RNAs	7	507	4	0.000799	0.00174
Downstream signaling of activated FGFR1	139	10.1	21	0.000955	0.00191
Downstream signaling of activated FGFR2	139	10.1	21	0.000955	0.00191
Downstream signaling of activated FGFR3	139	10.1	21	0.000955	0.00191
Downstream signaling of activated FGFR4	139	10.1	21	0.000955	0.00191
NGF signalling via TRKA from the plasma membrane	189	13.7	26	0.00107	0.0021
Nuclear import of Rev protein	31	2.25	8	0.00128	0.00241
RNA Polymerase I Transcription Termination	31	2.25	8	0.00128	0.00241
Insulin receptor signalling cascade	87	6.3	15	0.0013	0.00241
Signaling by TGF-beta Receptor Complex in Cancer	8	0.58	4	0.00151	0.00275
Mitotic G1-G1/S phases	126	9.13	19	0.00168	3
Cellular response to heat stress	72	5.22	13	0.00175	0.00307
Interactions of Rev with host cellular proteins	33	2.39	8	0.00197	0.0034
Prolonged ERK activation events	20	1.45	6	0.00226	0.00383
Signalling by NGF	273	19.8	33	0.00231	0.00385
RNA Polymerase I Transcription	34	2.46	8	0.00242	0.00395
Signaling by Insulin receptor	111	8.04	17	0.00245	0.00395
IRS-related events triggered by IGF1R	84	6.09	14	0.0026	0.00409
Innate Immune System	569	41.2	59	0.00262	0.00409
DNA Repair	141	10.2	20	0.00272	0.00415
DAP12 interactions	171	12.4	23	0.00274	0.00415
Signaling by Type 1 Insulin-like Growth Factor 1 Receptor (IGF1R)	87	6.3	14	0.00363	0.00534
IGF1R signaling cascade	87	6.3	14	0.00363	0.00534
Developmental Biology	438	31.7	47	0.00369	0.00535
Regulation of the Fanconi anemia pathway	10	725	4	0.00402	0.00574
Signaling by PDGF	177	12.8	23	0.00425	0.00596
Influenza Life Cycle	117	8.48	17	0.00429	0.00596
ARMS-mediated activation	16	1.16	5	0.00437	0.00599
IRS-mediated signalling	80	5.8	13	0.00457	0.00618
Cyclin D associated events in G1	30	2.17	7	0.00472	0.00621
G1 Phase	30	2.17	7	0.00472	0.00621
Signaling by the B Cell Receptor (BCR)	190	13.8	24	0.00509	0.00661
Regulatory RNA pathways	100	7.25	15	0.00526	0.00674
IRS-related events	82	5.94	13	0.00567	0.0071
Rev-mediated nuclear export of HIV RNA	31	2.25	7	0.00573	0.0071
Inflammasomes	17	1.23	5	0.00583	0.0071
Diseases of signal transduction	235	17	28	0.00591	0.0071
Membrane binding and targetting of GAG proteins	11	797	4	0.00596	0.0071
Synthesis And Processing Of GAG, GAGPOL Polyproteins	11	797	4	0.00596	0.0071
Influenza Infection	122	8.84	17	0.00658	0.00774
Frs2-mediated activation	18	1.3	5	0.0076	0.00884
mTORC1-mediated signalling	12	869	4	0.00843	0.00958
The NLRP3 inflammasome	12	869	4	0.00843	0.00958

Interferon Signaling	177	12.8	22	0.00861	0.00967
Late Phase of HIV Life Cycle	126	9.13	17	0.00906	0.00996
Host Interactions of HIV factors	126	9.13	17	0.00906	0.00996
Regulation of mRNA stability by proteins that bind AU-rich elements	87	6.3	13	0.00935	0.0102
Negative regulation of FGFR2 signaling	34	2.46	7	0.0097	0.0102
Signaling by NODAL	19	1.38	5	0.00971	0.0102
Pre-NOTCH Transcription and Translation	19	1.38	5	0.00971	0.0102
G2/M Checkpoints	51	3.7	9	0.0101	0.0104
PI3K Cascade	69	5	11	0.0101	0.0104
TGFBR1 KD Mutants in Cancer	7	507	3	0.0106	0.0107
Loss of Function of TGFBR1 in Cancer	7	507	3	0.0106	0.0107
Disease	669	48.5	64	11	11
