# Supplementary material for: Cells of the Maternal–Fetal Interface May Contribute to Epidural-Related Maternal Fever After Administration of Ropivacaine: The Role of Phosphatases DUSP9 and PHLPP1
Source: Int J Mol Sci. 2025 Jun 9;26(12):5520. doi: 10.3390/ijms26125520 (PMC12193418; doi:10.3390/ijms26125520)

Supplemental Data 3: Negative controls for Figure 3 (in main text)

Immunofluorescence staining of first trimester placental villi (first row) and decidua basalis (second row) from placental explant cultures (9th week of pregnancy) using Isotype control antibodies. Left panel: DAPI staining to visualize nuclei. Middle panel: Isotype Control for DUSP9 and PHLPP with anti-rabbit 488 secondary antibody (green). Right panel: Isotype control for HLA-G in combination with anti-mouse 568 secondary antibody (red). Bars indicate 50 µm. DB, decidua basalis; pV, placental villus;

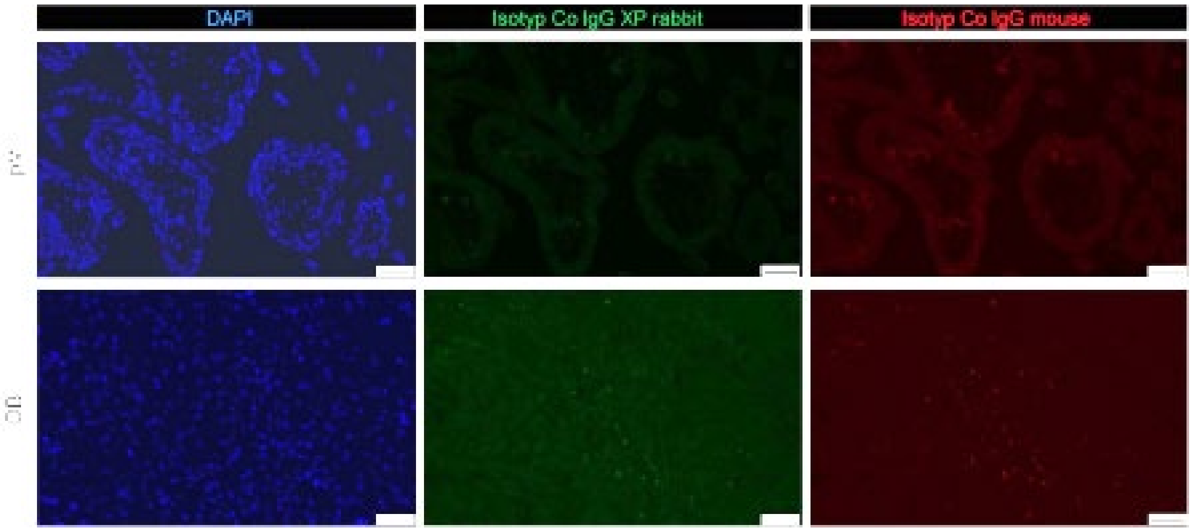

Supplement: Supplementary file 1 [file ijms-26-05520-s001.zip › ijms-3620049-supplementary/Supplementary Material/Supplementary Material S3.pdf]
